# Leveraging Student Volunteers to Connect Patients with Social Risk to Resources On a Coordinated Care Platform: A Case Study with Two Endocrinology Clinics

**DOI:** 10.5334/ijic.7633

**Published:** 2024-02-14

**Authors:** Grace Lee, Rebecca Liu, Eugenia R. McPeek Hinz, Janet Prvu Bettger, John Purakal, Susan E. Spratt

**Affiliations:** 1 Trinity College of Arts & Sciences, Duke University, Durham, North Carolina, USA; 2 Duke University Health System, Durham, North Carolina, USA; 3 Duke-Margolis Center for Health Policy, Duke University, Durham, North Carolina, USA; 4 Department of Health and Rehabilitation Sciences, Temple University, Philadelphia, Pennsylvania, USA; 5 Department of Emergency Medicine, Duke University School of Medicine, Durham, North Carolina, USA; 6 Samuel Dubois Cook Center on Social Equity, Duke University, Durham, North Carolina, USA; 7 Division of Endocrinology, Metabolism, and Nutrition, Department of Medicine, Duke University School of Medicine, Durham, North Carolina, USA; 8 Duke Population Health Management Office, Duke University School of Medicine, Durham, North Carolina, USA

**Keywords:** Social Drivers of Health, Social Resource Allocation, Student Volunteers, Health Related Social Needs, Coordinated Care Platform

## Abstract

**Introduction:**

Although unmet social needs can impact health outcomes, health systems often lack the capacity to fully address these needs. Our study describes a model that organized student volunteers as a community-based organisation (CBO) to serve as a social referral hub on a coordinated social care platform, NCCARE360.

**Description:**

Patients at two endocrinology clinics were systematically screened for social needs. Patients who screened positive and agreed to receive help were referred via NCCARE360 to student ‘Help Desk’ volunteers, who organised as a CBO. Trained student volunteers called patients to place referrals to resources and document them on the platform. The platform includes documentation at several levels, acting as a shared information source between healthcare providers, volunteer student patient navigators, and community resources. Navigators followed up with patients to problem-solve barriers and track referral outcomes on the platform, visible to all parties working with the patient.

**Discussion:**

Of the 44 patients who screened positive for social needs and were given referrals by Help Desk, 41 (93%) were reached for follow-up. Thirty-six patients (82%) connected to at least one resource. These results speak to the feasibility and utility of organising undergraduate student volunteers into a social referral hub to connect patients to resources on a coordinated care platform.

**Conclusion:**

Organising students as a CBO on a centralized social care platform can help bridge a critical gap between healthcare and social services, addressing health system capacity and ultimately improving patients’ connections with resources.

## Introduction

Social drivers of health (SDOH) are defined as the “conditions in which people are born, grow, live, work and age” by the World Health Organization [1]. These conditions play a significant role in people’s health and well-being for either good or detriment. SDOH include but are not limited to food security, housing status, access to transportation, and the physical environment. Addressing SDOH is vital to improving health and reducing health disparities on a population level. Healthcare systems have increasingly dedicated resources toward addressing SDOH, often by implementing screening protocols during primary and specialty care clinic visits [2,3,4,5,6,7,8]. However, health systems often lack the capacity for social service referral and follow-up for patients after needs are identified [9,10]. As a result, even when referrals are made, follow-up is limited and outcomes are often unknown [11,12].

Studies in the United States have found success using trained students to serve as patient navigators who refer and provide follow-up with patients to ensure connection to resources [13,14,15,16]. Although students can bring value to the process of integrating health and social care, as volunteers they are unable to access private electronic health records (EHR) to facilitate information transfer and continuity of care. In our study, we addressed this documentation issue by establishing a university student volunteer organisation called Help Desk as a community-based organisation (CBO). As a CBO, Help Desk registered to be listed on a statewide coordinated care platform. The statewide platform, NCCARE360, was initiated in 2019 by the North Carolina Department of Health and Human Services to serve as a repository of community resources and allow users, including CBOs, to place and track electronic referrals for patients with social needs [17,18,19]. Importantly, referrals and their outcomes are visible to all parties with access to the platform, including providers and CBOs [17]. As a CBO, Help Desk offered a potential solution to increase health system capacity for social support and integrated care by serving as a hub between healthcare providers and other community and social services. The volunteers function as navigators, calling patients with identified needs, providing referrals to specific services, and using NCCARE360 for transparency across parties to problem-solve connecting to resources.

This report describes a case study from two endocrinology clinics that are part of a larger evaluation effort of integrated health and social care for a health system in the Southeastern United States. The model organized student volunteers into a CBO, allowing students to receive patient referrals from healthcare providers, support patients with unmet social needs to connect to other CBOs and public services, and track patient-resource connection outcomes via a statewide referral platform.

## Ethical approval

This study was approved by the institutional review board for the Duke University academic medical center.

## Implementation Background

### Study Setting

Our case study examined SDOH systematic screening and resource referral through Help Desk at two ambulatory endocrinology clinics. These clinics are part of a quaternary care academic institution and are located in two counties in central North Carolina in the United States. Both counties are ethnically diverse, with Black, Hispanic, Asian, and other minority groups composing 56.6% and 41.2%, respectively, of each county’s population [20,21].

The Help Desk volunteer team comprised university undergraduate students from a private university that is also home to a nationally prominent academic medical center. The volunteer team consisted of five students, two of whom served as coordinators on the leadership team.

### Help Desk Organisation and Volunteers

Help Desk is a student-led undergraduate initiative to address unmet social needs and reduce health disparities [14,22]. When the model was first developed in 2018, Help Desk volunteers provided follow-up phone calls for case managers at a federally qualified health center. Case managers screened patients using the Protocol for Responding to and Assessing Patients’ Assets, Risks, and Experiences (PRAPARE) tool, and referred them to CBOs to address identified unmet social needs [14,22,23]. The success of the Help Desk model led to its expansion to the affiliated Emergency Department and two Endocrinology clinics. In these clinics, volunteers are leveraged in various ways: 1) to screen patients for social needs, 2) to refer patients to community resources, and/or 3) to follow up with patients already referred to resources by clinical personnel (and/or Help Desk) to assess whether connection was made and troubleshoot barriers to ensure successful connection.

Help Desk is an official university-recognized organisation. Students complete over 20 hours of training on SDOH; motivational interviewing; local, state, and federal resources; privacy laws and expectations according to the Health Insurance Portability and Accountability Act (HIPAA); protocols for calling patients through shadowing and reverse shadowing calls; and cultural humility [24]. The leadership team comprises undergraduate students ranging from freshmen to seniors. Roles include program coordinators, who oversee program expansion; site coordinators, who oversee the day-to-day operations at each clinic; recruitment and training chairs, who are responsible for overseeing volunteer onboarding and training; and NCCARE360 liaisons, who communicate feedback about the platform to various stakeholders. Student leadership is guided by faculty advisors and clinic leadership in developing workflows, scripts, and escalation protocols that serve each clinic’s needs and complement the clinic’s available resources.

### NCCARE360

NCCARE360 is an electronic social care technology platform developed by Unite Us in collaboration with the North Carolina Department of Health and Human Services, and two nonprofits, United Way and the Foundation for Health Leadership and Innovation [17,18,19,25]. Its goal is to serve as a statewide resource directory of CBOs and social, behavioral, and government resources to provide a closed-loop format for referrals to track outcomes. A variety of CBOs, other connecting resources, and supportive individuals, including community health workers, assist connection of individuals to health-related social needs (HRSN) support.

After a referral is placed on NCCARE360, the recipient organisation can either accept or reject it. If the referral is accepted, the organisation receives an individual’s case and directly contacts the individual for further help. The responsibility of contact therefore lies with the referral organisation. In previous resource referral and follow-up models before NCCARE360’s implementation, when a resource referral was made, the responsibility of contact lay with the patient. After making contact, the recipient organisation documents all interactions with the patient and referral outcomes, i.e., whether they were able to enroll the patient or deliver services, on the platform. Referral outcomes and interactions are viewable for all parties with access to the platform, allowing cross-sector communication and keeping all stakeholders informed of the patient’s progress in addressing their social needs.

Although the vision of NCCARE360 is to provide a straightforward, accessible, and trackable social service network across the state, a patient navigator with dedicated time to help patients through the referral process remains key. Identifying this workforce is essential to addressing the HRSNs of our patients. In September 2022, we created a Help Desk CBO within NCCARE360 to act as a social navigation hub and refer patients to CBOs that could directly address the patient’s needs, such as food delivery services. Because the platform was embedded into the health system’s EHR in January 2021, providers could view referrals made by Help Desk, whether the patient connected to resources, and whether the patient’s social needs were addressed.

### Endocrinology and Student Help Desk Collaboration

The Division of Endocrinology, Metabolism, and Nutrition at the site of the study has four clinical sites that cared for 23,350 patients in 2021. The cost of managing diabetes is extensive, creating financial strain on patients [26,27,28]. Many patients are forced to choose between paying for medication, testing supplies, utilities, food, or rent. Food insecurity has also been correlated with hypoglycemia and hyperglycemia [26,27,28,29]. Due to these characteristics, we chose to pilot our model at two endocrine clinics that did not have access to social workers. To develop and implement this workflow, student leadership worked with faculty advisors, providers, physician assistants, and nurse managers to assess the best methods for screening patients and placing referrals.

By adding Help Desk as a CBO on NCCARE360, Endocrinology staff had a centralized navigation team ready to help connect patients to resources. Help Desk’s CBO status allowed for a relatively fluid way of connecting patients from providers to a social hub and served as a platform through which providers could view referral outcomes and notes made by Help Desk. Other models would require different documentation external to the EHR, such as a secure spreadsheet, which was used in previous Help Desk models.

## Methods

### Screening, Referral, and Follow-Up Workflow

**Figure 1:**
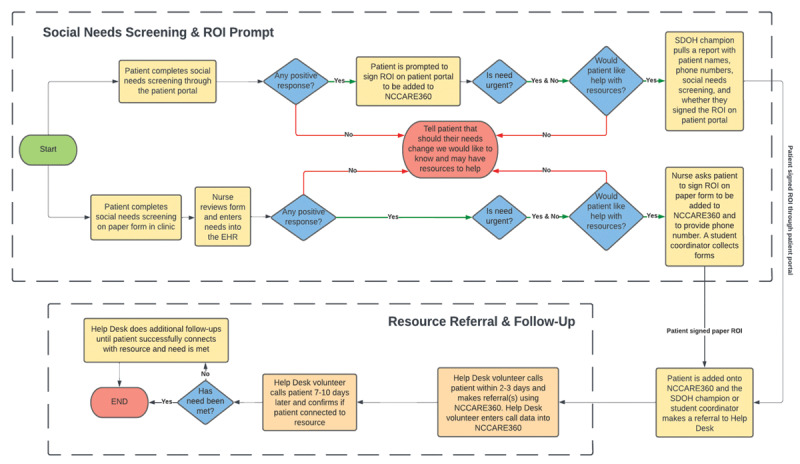
Endocrine HRSN screening and referral workflow.

Patients were screened through two methods: an online workflow through a patient portal and a paper form workflow. Via the portal, patients completed a social factors questionnaire sent prior to their clinic visit. If the patients screened positive, they were asked to sign a Release of Information (ROI) form that would allow them to be added to NCCARE360. They were then asked if they wanted help. A provider, titled the SDOH champion, would then run a query in the EHR to find patients who had indicated a social need, signed the portal ROI, and requested help (Figure 1). The SDOH champion would then refer the patient to Help Desk on NCCARE360.

In the paper screening workflow, patients filled out an HRSN screening questionnaire during check-in and signed a paper ROI if they screened positive for social needs and requested help. A student coordinator collected these forms, created a patient referral on NCCARE360, and sent the referral to Help Desk (Figure 1).

After a Help Desk referral was placed, a student coordinator accepted the referral, thus changing its status to an “open case,” and assigned patients to volunteers. The volunteer then called the patient within 2–3 days. If no initial contact was made, volunteers would attempt to contact the patient at least 3 times. During the referral call, the volunteer made referrals to other CBOs via NCCARE360. After the call, volunteers sent a summary text message detailing what referrals were placed. After 7–10 days, the volunteer completed a follow-up call to ensure the patient had connected to resources and that their needs were met. If needs were not met, the volunteer continued to provide telephonic outreach to the patient. For cases that needed to be escalated, such as in cases where the patient was in physical danger, volunteers reached out to the SDOH champion, who then connected to the patient’s healthcare team. Once the referral call and/or follow-up attempts had been exhausted, whether the patient was reached, the Help Desk case was categorized as “closed.”

Every patient interaction or call attempt was documented in the referral case on NCCARE360. Providers could see this information, case status, and eventual resolution by launching the platform through the EHR. This was a key advantage of making Help Desk an NCCARE360 CBO.

### Study Aim and Analysis

The goal of this study was to determine the feasibility of establishing student volunteers as a CBO on NCCARE360 to accept referrals from providers and then make resource referrals and conduct follow-up with those patients to ensure resource connection. Volunteers conducted social needs screening and referral calls for six months from September 2022 to March 2023.

To examine the feasibility of leveraging student volunteers to serve as a CBO and facilitate patient referral to resources, we evaluated (a) the number of successful connections between volunteer and patient for both referral and follow-up calls and (b) the rate of successful connection between patient and resource. We manually parsed through the referral outcomes and volunteer notes on NCCARE360 to determine both measures. We considered a patient connected to a resource if the connection was confirmed during a phone call by a volunteer, if the CBO had accepted the referral on NCCARE360 and stated that they had successfully contacted the patient, and/or if the CBO stated that they had invited the patient to access resources.

## Results

From August 1^st^, 2022, to March 26^th^, 2023, 9955 patients were seen across the two ambulatory care endocrine clinics (Table 1). A total of 7771 patients were screened for HRSNs with similar volumes by sex compared to the overall population. Food insecurity was found for 605 (7.8%) and transportation needs for 212 (2.7%) individuals across the total screened population. Of those individuals with HRSN, 151 signed the ROI, and 83 of these patients who requested help were referred to Help Desk. It was more common for females to accept referrals to NCCARE360 than males, and the average age of those who screened positive was younger than those who did not screen positive (Table 1).

**Table 1:** Demographics of HRSN Screening Overall, by Food Insecurity, Transportation Needs and Referral to Help Desk.


8/1/2022 – 3/26/2023	MALE	FEMALE	TOTALS	MEAN AGE

Population – unique patients (% of total)*	2983 (30.0%)	6971 (70.0%)	9955	56.9

**Screened** (% of total)	**2347 (30.2%)**	**5424 (69.8%)**	**7771** (78.1% of all patients)	**57.2**

Not Screened (% of total)	636 (29.1%)	1547 (70.8%)	2184	56.2

**Food Insecurity** (% of total)	**170 (28.1%)**	**435 (71.9%)**	**605** (7.8% of all patients screened)	**51.6**

No Food Needs	2042	4699	6741	57.6

**Transportation Needs** (% of total)	**51 (24.1%)**	**161 (75.9%)**	**212** (2.7% of all patients screened)	**55.1**

No Transportation Needs	2263	5176	7439	57.2

Accept Referral to NCCARE360 (% of total)	**33 (21.9%)**	**118 (78.1%)**	**151**	**50.5**

Decline	79	201	280	51.1


*1 patient’s sex was categorized as unknown. This individual screened negative for food insecurity and transportation needs.

Of the 83 patients who screened positive, requested help for social needs, and were referred to the Help Desk CBO, 64 cases were closed, i.e., either outcomes were determined, or all call attempts were completed without connecting to the patient. The remaining cases (N = 19) are open cases, meaning that volunteers are still completing their call attempts. Out of closed cases, volunteers reached 52 patients at least once, placing referrals in 44 cases. Patients who were not referred by Help Desk (N = 8) included those whose social needs had been resolved before the referral call, patients who spoke a language other than English, and patients who had been sent to another referral organisation prior to the call.

**Figure 2:**
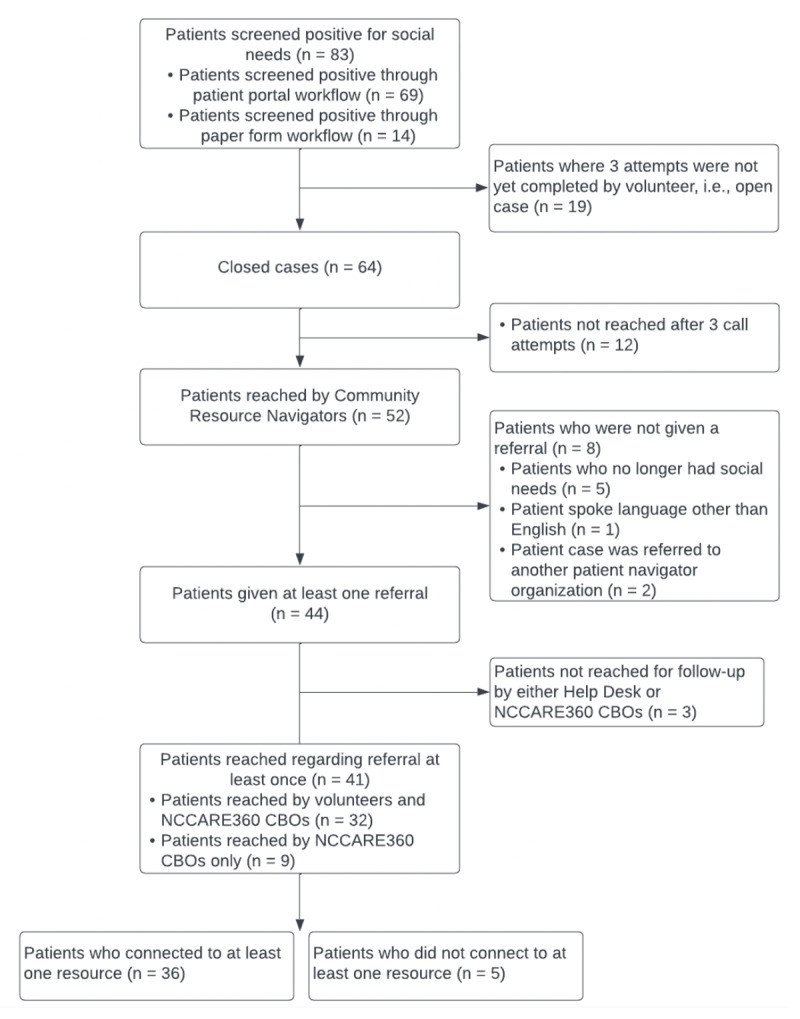
Flow diagram of patient interactions and outcomes.

For cases where a referral was placed (N = 44), 41 (93%) patients were reached for follow-up by either Help Desk and an NCCARE360 CBO that Help Desk had referred the patient to (N = 32) or an NCCARE360 CBO only (N = 9). Thirty-six (82%) patients connected to at least one resource. Only 5 patients did not connect to resources during follow-up. These patients did not connect to resources because their referral was rejected by the CBO due to not being eligible for the specific service, and there were no additional resources available given their location and specific need. Figure 2 provides a flow diagram of outcomes for Help Desk referrals.

**Figure 3:**
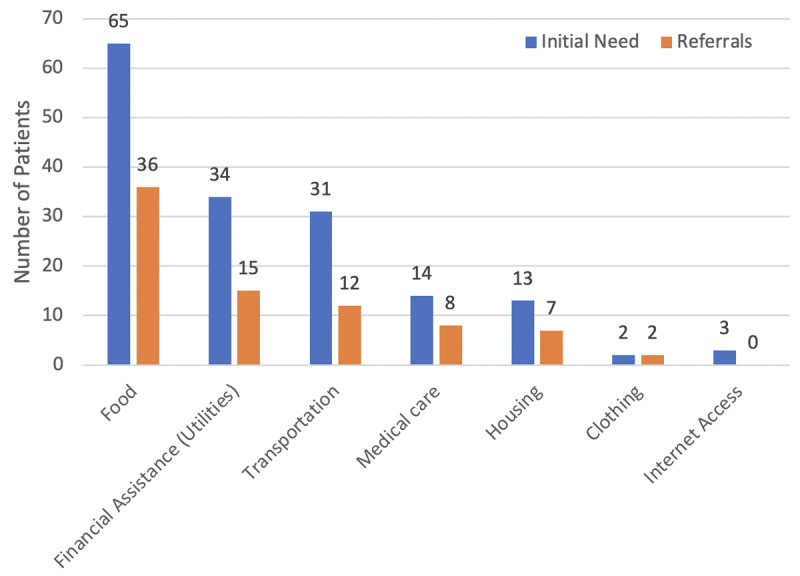
Graph depicting domains of patient needs and referrals made by volunteers between September 1^st^, 2022, and March 26^th^, 2023.

The most common social needs and referrals were food, transportation, and financial assistance for utilities and other costs. Ninety-four percent of patients reported at least one need in these domains (Figure 3). Other reported social needs included medical care, housing, and clothing. Numbers include both open and closed cases.

## Discussion

Screening and addressing social needs are essential steps toward improving health equity [3,4,5,6,7,8,9]. Interventions addressing SDOH on a population level can be supported by coordinated care platforms that connect providers, patient navigators, and CBOs. Implementing workflows through which these platforms can be used to serve patients requires cross-sector collaboration between patient navigators, healthcare systems, private companies, governments, and CBOs.

Our case study demonstrates how a CBO of student volunteers can be leveraged as a resource referral hub. Of 64 closed cases, over 81% of patients (n = 52) were contacted by volunteers at least once. Of the 44 patients given referrals, 93% of patients were reached for follow-up either by Help Desk, a CBO, or both, which is comparable to or greater than the rate found in previous interventions [16,22,30,31]. Of the patients who had received a referral from a Help Desk volunteer, 82% connected to at least one community resource. Similar to the rate of successful follow-up, this connection rate is greater than many other interventions in the literature, including the original Help Desk pilot study, where patients had a 48% connection rate to resources [14,16,22,30,31].

We believe that our greater connection rates are in large part due to the combined efforts of volunteer navigators and their use of the NCCARE360 coordinated care platform. By placing the onus of reaching out on the CBO, not the patient, NCCARE360 addresses many major barriers to resource connection reported by patients in previous interventions, such as missing CBO contact information and lack of time to reach out to CBOs [14,31]. In addition, in previous interventions, if a patient could not be reached for follow-up by the person who made the referral, their outcomes were often unknown [12,13]. Our follow-up and patient-to-resource connection rate was likely higher, therefore, because integrating navigators to make full use of the platform (a) addressed patient-resource connection barriers such as missing contact information and (b) supported documentation and communication between providers, patient navigators and other referral organisations, and community resources on the interactions and recorded outcomes between CBOs and patients. Platform use thus enabled greater integration of care. Ultimately, these results speak to the feasibility of having students serve as a CBO and leverage a statewide, centralized platform for resource referral and outcome tracking.

To promote integration between healthcare systems, patient navigators, and CBOs, Unite Us is expanding and creating similar coordinated care networks across the United States. At present, 44 of 50 states are implementing care networks [32]. Similar initiatives to build digital platforms that facilitate cross-sector communication exist across the globe, including in Switzerland, Belgium, and the United Kingdom [33,34]. In the United Kingdom, private companies have partnered with healthcare systems and CBOs to develop centralized platforms that, like NCCARE360, allow organisations to send referrals to non-clinical services and track outcomes on a central platform [35,36]. In Estonia, there is an integrated care reform initiative to create a centralized system of social, medical, and vocational support services to support patient populations [37]. We hope that our study can inform organisations in other regions about potential workflows through which coordinated care networks can be used to support social needs screening and referral.

### Hurdles to Implementation

Implementation required overcoming several hurdles, including generating buy-in to use the platform itself, obtaining a signed ROI, difficulty in reaching patients at mutually convenient times, and changing resource availability.

One barrier to using NCCARE360 was hesitancy by providers to use a system that often did not provide quick follow-up. However, because of the sizable number of undergraduate students eager to learn more about healthcare and assist patients, we could maintain a consistent workforce, ensure that all patients were referred and followed up with in a timely manner, and document patients’ social care journeys. Help Desk volunteers were also able to call patients during evenings and weekends, when more patients may be off work, compared to paid employees who usually work during standard business hours. Through the program, patients received help for their social needs and learned more about the available national, state, and local community resources. Likewise, volunteers developed patient interaction skills and gained an understanding of SDOH [14,24].

To protect patient information, a signed ROI to use NCCARE360 was required by HIPAA regulations. By making Help Desk a CBO on the platform, the burden of obtaining the ROI was placed on the sender of the referral. If a patient screened positive for social needs via the patient portal or paper form in-clinic and requested further help, they were prompted to sign and complete an ROI form on the patient portal or on paper, respectively. After this step, the patient was added onto NCCARE360, and a referral could be made to Help Desk. However, if the patient did not complete the ROI, a referral could not be made, thus limiting Help Desk’s outreach to patients.

Given that Help Desk volunteer interaction with patients was completely telephonic, successful contact with a patient was dependent on whether the volunteer called the patient at a mutually convenient time. The inability to find a mutually convenient time limited Help Desk’s ability to address patient needs. However, efforts were made to overcome this obstacle by establishing protocols for volunteers in the event that they could not reach a patient. These protocols included leaving voicemails, calling back at least 3 times on different days and at different times, and providing the patient with Help Desk’s contact information. Ideally, patients with social needs should be helped on-site on the day of the clinical encounter, but most clinical areas do not employ onsite social workers or community health workers.

Due to fluctuating economic conditions and a high prevalence of social needs, resources were often underfunded and would pause referrals on the platform. As a result, certain needs, such as housing and utilities assistance, could not be met by the available CBOs in the area. Our volunteers navigated this hurdle as best as possible, finding other resources and sharing successful referrals during weekly case reviews.

### Limitations

The study and model had a few limitations, including being limited to English-speaking volunteers and patients and the lack of bidirectional flow between NCCARE360 and the EHR. In addition, most medical centers are not linked to academic centers, limiting the implementation of a workflow using undergraduate students. However, our workflow could be replicated using other social resource connectors, such as community health workers and organisations acting as referral hubs that already exist on the platform.

For this study, we were only able to recruit English-speaking volunteers. We were unable to reach one patient due to this language barrier. To address this issue, our upcoming volunteer cohort includes multiple Spanish-speaking students, of which one has been assigned to call endocrine patients. In addition, our paper form is only in English, though we plan to include a Spanish version once the cohort is expanded.

The lack of bidirectional flow to the EHR also presented a challenge. Neither documentation in the EHR nor NCCARE360 is bidirectional. Therefore, documentation is only viewable by health system users if they know the referral in NCCARE360 has been placed. To address this challenge, we established rigid volunteer documentation protocols which allowed patient updates to be clearly communicated to providers in the event that they would like to determine whether a patient’s needs have been addressed. However, bidirectional flow would allow for more seamless cross-sector collaboration and communication.

Because our model was implemented in an urban area, this case study may not be generalizable to other communities, such as those in rural areas without academic medical centres or where access to resources might be more limited. In addition, data collection was highly dependent upon volunteer and CBO self-reported notes on patient interactions and referrals. When the workflow was first implemented, for example, there were discrepancies among volunteers on delineating what was considered a “resolved” case. When volunteers could not contact patients for a final follow-up call, some cases were marked as “resolved” due to confirmation from a CBO about patient connection whereas other cases were marked as “unresolved” due to loss of patient contact. To minimize the risk of erroneous or vague reporting, all volunteers underwent significant training on the implemented workflow and NCCARE360, and discrepancies were clarified and corrected during weekly case reviews.

Deploying a Help Desk intervention required significant cooperation between healthcare administrators and student leadership. Structured training on topics such as local resources, motivational interviewing, and HIPAA remains key to all workflows [24]. The intervention described in this case study is part of a greater effort to create a centralized social support system for patients, and our findings demonstrate the value of having students serve as a key connector for patients to resources to increase health system capacity for social support. Our findings also speak to the contribution of coordinated care platforms in increasing patient-resource connection rates and facilitating cross-sector communication to address social needs.

## Lessons Learned

The following principles were integral in establishing Help Desk as a CBO on NCCARE360: 1) Collaboration with a pre-existing student group interested in addressing social needs, 2) Realistic goals and workload, 3) Comprehensive training, and 4) Frequent and structured communication between all stakeholders.

1. Prior to its establishment as a CBO on NCCARE360, Help Desk was already an established undergraduate organisation. Undergraduate universities often contain hundreds or thousands of pre-health students who are eager to gain experience and help. Utilizing this enthusiasm aided in establishing a reliable and driven volunteer base, thus allowing for effective operation as a CBO. An organized leadership structure was also integral in the implementation process. The Help Desk leadership team consists of program coordinators, site coordinators, recruitment and training chairs, NCCARE360 liaisons, and programming chairs, who together manage volunteers and keep the organisation accountable to all patients and clinic partners.
2. An important contributor to the success of this case was setting realistic goals for student volunteers to become accustomed to patient interactions and the workflow. We found that starting with two clinics was beneficial in establishing Help Desk as a CBO. This initial scale allowed students to gain confidence in calling patients and making referrals. It also allowed the workflow to be fine-tuned according to clinic needs.
3. Comprehensive training was essential in establishing knowledgeable and skilled volunteers. Help Desk volunteers were required to undergo extensive training on HRSN; local, state, and federal resources; motivational interviewing; and privacy laws and expectations (HIPAA). Volunteers were also briefed on protocols for calling patients as well as escalation protocols for situations including intimate partner violence, suicide, or imminent eviction. For further preparation and to assess efficacy, volunteers were required to complete practice calls, shadowing of calls made by current volunteers, and reverse shadowing, where current volunteers provided new volunteers with feedback and guidance during calls.
4. Frequent communication was required to build collaborative capacity, troubleshoot barriers, and discuss changes in the platform and workflows. Developing and implementing a workflow that served each clinic’s needs and complemented the clinic’s available resources required weekly meetings between volunteers and student leadership and between student leadership and clinic leadership. Meetings were done on an as-needed basis between both leadership teams with state and private entities involved in the implementation of NCCARE360.

## Conclusion

There is a need for a patient navigator role to facilitate connection to social resources for patients with social needs. Clinical staff are not trained in resource allocation and often do not have the time for follow-up, particularly when the referral is rejected or not resolved. Help Desk student volunteers can act as patient navigators, much like community health workers, to fill this gap.

Patient referral in this workflow involved the use of NCCARE360, a centralized social care platform. As a CBO on NCCARE360, Help Desk can document their interactions and referral outcomes in a manner that is viewable to providers and other groups using the platform. Volunteers and providers are also able to view referral resolutions documented by CBOs, closing the loop from referral to outcome. Leveraging students to act as the key connector between patients and resources is one workflow through which these platforms can be used to address social needs and enable greater integration of care.

## References

[1] Social Determinants of Health [Internet]. World Health Organization. [cited 2023 Mar 25]. Available from: http://www.who.int/social_determinants/en/.

[2] Bleacher H, Lyon C, Mims L, Cebuhar K, Begum A. The Feasibility of Screening for Social Determinants of Health: Seven Lessons Learned. Fam Pract Manag. 2019. October; 26(5): 13–9. [cited 2023 Mar 26]. Available from: https://www.aafp.org/pubs/fpm/issues/2019/0900/p13.html. 31502814

[3] LaForge K, Gold R, Cottrell E, Bunce AE, Proser M, Hollombe C, et al. How 6 Organisations Developed Tools and Processes for Social Determinants of Health Screening in Primary Care: An Overview. Journal of Ambulatory Care Management [Internet]. 2018; 41(1): 2–14. [cited 2023 Mar 23]. DOI: 10.1097/JAC.0000000000000221 28990990 PMC5705433

[4] Gold R, Cottrell E, Bunce A, Middendorf M, Hollombe C, Cowburn S, et al. Developing Electronic Health Record (EHR) Strategies Related to Health Center Patients’ Social Determinants of Health. J Am Board Fam Med [Internet]. 2017. July 1; 30(4): 428–47. [cited 2023 Mar 23]. DOI: 10.3122/jabfm.2017.04.170046 28720625 PMC5618800

[5] Gottlieb L, Tobey R, Cantor J, Hessler D, Adler NE. Integrating Social And Medical Data To Improve Population Health: Opportunities And Barriers. Health Affairs [Internet]. 2016. November 1; 35(11): 2116–23. [cited 2023 Mar 26]. DOI: 10.1377/hlthaff.2016.0723 27834254

[6] O’Gurek DT, Henke C. A Practical Approach to Screening for Social Determinants of Health. FPM [Internet]. 2018. June; 25(3): 7–12. [cited 2023 Mar 23]. Available from: https://www.aafp.org/fpm/2018/0500/p7.html. 29989777

[7] Fiori K, Patel M, Sanderson D, Parsons A, Hodgson S, Scholnick J, et al. From policy statement to practice: Integrating Social Needs Screening and referral assistance with community health workers in an urban academic health center. Journal of Primary Care & Community Health. 2019; 10: 215013271989920. [cited 2023 Mar 26]. DOI: 10.1177/2150132719899207 PMC6940600 31894711

[8] Drake C, Batchelder H, Lian T, et al. Implementation of social needs screening in primary care: A qualitative study using the Health Equity Implementation Framework; 2021. [cited 2023 Mar 26]. DOI: 10.21203/rs.3.rs-455846/v1 PMC8445654 34530826

[9] Young RA, Burge SK, Kumar KA, Wilson JM, Ortiz DF. A Time-Motion Study of Primary Care Physicians’ Work in the Electronic Health Record Era. Fam Med. 2018; 50(2): 91–99. [cited 2023 Mar 26]. DOI: 10.22454/FamMed.2018.184803 29432623

[10] De Marchis E, Pantell M, Fichtenberg C, Gottlieb LM. Prevalence of Patient-Reported Social Risk Factors and Receipt of Assistance in Federally Funded Health Centers. J Gen Intern Med. 2020; 35(1): 360–364. DOI: 10.1007/s11606-019-05393-w 31677105 PMC6957636

[11] Gottlieb LM, Wing H, Adler NE. A Systematic Review of Interventions on Patients’ Social and Economic Needs. American Journal of Preventive Medicine [Internet]. 2017. November; 53(5): 719–29. [cited 2023 Mar 17]. DOI: 10.1016/j.amepre.2017.05.011 28688725

[12] Bickerdike L, Booth A, Wilson PM, Farley K, Wright K. Social prescribing: less rhetoric and more reality. A systematic review of the evidence. BMJ Open [Internet]. 2017. April 1 [cited 2019 Mar 23]; 7(4): e013384. DOI: 10.1136/bmjopen-2016-013384 PMC5558801 28389486

[13] Sandhu S, Xu J, Eisenson H, Prvu Bettger J. Workforce models to screen for and address patients’ unmet social needs in the clinic setting: A scoping review. Journal of Primary Care & Community Health. 2021; 12: 215013272110210. [cited 2023 Mar 17]. DOI: 10.1177/21501327211021021 PMC8772357 34053370

[14] Sandhu S, Xu J, Blanchard L, Eisenson H, Crowder C, Munoz VS, Drake C, Bettger JP. A community resource navigator model: utilizing student volunteers to integrate health and social care in a community health center setting. International Journal of Integrated Care. 2021 Jan; 21(1). [cited 2023 Mar 17]. DOI: 10.5334/ijic.5501 PMC7863845 33597833

[15] Losonczy LI, Hsieh D, Wang M, Hahn C, Trivedi T, Rodriguez M, et al. The Highland Health Advocates: A preliminary evaluation of a novel programme addressing the social needs of emergency department patients. Emergency Medicine Journal. 2017; 34(9): 599–605. [cited 2023 Mar 17]. DOI: 10.1136/emermed-2015-205662 28642372

[16] Herrera T, Fiori KP, Archer-Dyer H, Lounsbury DW, Wylie-Rosett J. Social Determinants of health screening by preclinical medical students during the COVID-19 pandemic: Service-Based Learning Case Study. JMIR Med Educ. 2022 Jan 17; 8(1): e32818. [cited 2023 Mar 17]. DOI: 10.2196/32818 35037885 PMC8804950

[17] UNITE US: Cross-sector collaboration software; powered by community [Internet]. uniteus.com. 2023 [cited 2023 Mar 30]. Available from: https://uniteus.com/.

[18] NCCARE360 [Internet]. NCDHHS. [cited 2023 Mar 30]. Available from: https://www.ncdhhs.gov/about/department-initiatives/healthy-opportunities/nccare360.

[19] Thomas A, Ferguson E. NCCARE360: building healthier communities through collaboration. North Carolina medical journal. 2019 Sep 1; 80(5): 308. [cited 2023 Mar 17]. DOI: 10.18043/ncm.80.5.308 31471517

[20] U.S. Census Bureau quickfacts: Durham County North Carolina [Internet]. [cited 2023 Mar 31]. Available from: https://www.census.gov/quickfacts/durhamcountynorthcarolina.

[21] U.S. Census Bureau quickfacts: Wake County, North Carolina [Internet]. [cited 2023 Mar 31]. Available from: https://www.census.gov/quickfacts/wakecountynorthcarolina.

[22] Lian T, Kutzer K, Gautam D, Eisenson H, Crowder JC, Esmaili E, et al. Factors associated with patients’ connection to referred social needs resources at a Federally Qualified Health Center. Journal of Primary Care & Community Health. 2021; 12: 215013272110243. [cited 2023 Mar 17]. DOI: 10.1177/21501327211024390 PMC8202269 34120507

[23] Weir RC, Proser M, Jester M, Li V, Hood-Ronick CM, Gurewich D. Collecting Social Determinants of Health Data in the Clinical Setting: Findings from National PRAPARE Implementation. J Health Care Poor Underserved. 2020; 31(2): 1018–1035. [cited 2023 Mar 17]. DOI: 10.1353/hpu.2020.0075 33410822

[24] Gautam D, Kutzer K, Sandhu S, Dennis E, Xu J, Blanchard L, Munoz V, Drake C, Crowder C, Eisenson H, Bettger J. Training Student Volunteers as “Community Resource Navigators” to Integrate Health and Social Care in Primary Care. International Journal of Integrated Care. 2022 Apr 8; 22(S1). [cited 2023 Mar 17]. DOI: 10.5334/ijic.ICIC21135

[25] Wortman Z, Tilson EC, Cohen MK. Buying Health For North Carolinians: Addressing Nonmedical Drivers Of Health At Scale: This article describes initiatives the North Carolina Department of Health and Human Services is implementing to integrate medical and nonmedical drivers of health. Health Affairs. 2020 Apr 1; 39(4): 649–54. [cited 2023 Mar 17]. DOI: 10.1377/hlthaff.2019.01583 32250668

[26] Mendoza JA, Haaland W, D’Agostino RB, Martini L, Pihoker C, Frongillo EA, Mayer-Davis EJ, Liu LL, Dabelea D, Lawrence JM, Liese AD. Food insecurity is associated with high-risk glycemic control and higher health care utilization among youth and young adults with type 1 diabetes. Diabetes research and clinical practice. 2018 Apr 1; 138: 128–37. [cited 2023 Mar 17]. DOI: 10.1016/j.diabres.2018.01.035 29427695 PMC5910177

[27] Berkowitz SA, Baggett TP, Wexler DJ, Huskey KW, Wee CC. Food insecurity and metabolic control among US adults with diabetes. Diabetes care. 2013 Oct 1; 36(10): 3093–9. [cited 2023 Mar 17]. DOI: 10.2337/dc13-0570 23757436 PMC3781549

[28] Seligman HK, Jacobs EA, López A, Tschann J, Fernandez A. Food insecurity and glycemic control among low-income patients with type 2 diabetes. Diabetes care. 2012 Feb 1; 35(2): 233–8. [cited 2023 Mar 17]. DOI: 10.2337/dc11-1627 22210570 PMC3263865

[29] Reid LA, Zheng S, Mendoza JA, Reboussin BA, Roberts AJ, Sauder KA, Lawrence JM, Jensen E, Henkin L, Flory K, Knight LM. Household Food Insecurity and Fear of Hypoglycemia in Adolescents and Young Adults With Diabetes and Parents of Youth With Diabetes. Diabetes Care. 2022 Jun 30; [cited 2023 Mar 17]. DOI: 10.2337/dc21-1807 PMC9887608 35771776

[30] De Marchis E, Pantell M, Fichtenberg C, Gottlieb LM. Prevalence of patient-reported social risk factors and receipt of assistance in federally funded health centers. Journal of General Internal Medicine. 2019; 35(1): 360–4. [cited 2023 Mar 17]. DOI: 10.1007/s11606-019-05393-w 31677105 PMC6957636

[31] Sandhu S, Lian T, Smeltz L, Drake C, Eisenson H, Bettger JP. Patient barriers to accessing referred resources for unmet social needs. The Journal of the American Board of Family Medicine. 2022 Jul 1; 35(4): 793–802. [cited 2023 Mar 17]. DOI: 10.3122/jabfm.2022.04.210462 35896446

[32] Networks [Internet]. uniteus.com. 2023 [cited 2023 Mar 30]. Available from: https://uniteus.com/networks/.

[33] Sandhu S, Sharma A, Cholera R, Bettger JP. Integrated Health and Social Care in the United States: A decade of policy progress. International Journal of Integrated Care. 2021; 21(4). [cited 2023 Mar 17]. DOI: 10.5334/ijic.5687 PMC8570194 34785994

[34] de Almeida Mello J, Wellens NIH, Hermans K, De Stampa M, Cerase V, Vereker N, et al. The implementation of Integrated Health Information Systems – Research Studies from 7 countries involving the INTERRAI Assessment System. International Journal of Integrated Care. 2023; 23(1). [cited 2023 Mar 17]. DOI: 10.5334/ijic.6968 PMC9936911 36819613

[35] Pdh.platform [Internet]. Priority Digital Health. [cited 2023 Mar 30]. Available from: https://www.prioritydigitalhealth.com/platform.

[36] Partners: Elemental social prescription connector [Internet]. EMIS. [cited 2023 Mar 30]. Available from: https://www.emishealth.com/partners/social-prescribing-tools/elemental-social-prescription-connector#enquiry.

[37] Meyer I, Aavik-Märtmaa G, Poppe A, Müller S, Lewis L, Terris A, et al. Drilling for ‘New Oil’ in Care Integration – Co-production of the concept and specification of an Integrated Data Centre for Policy Decision Making, care planning, and research in estoni. International Journal of Integrated Care. 2023; 23: 17. [cited 2023 Mar 17]. DOI: 10.5334/ijic.6953 PMC10064888 37006718

